# Study protocol: HepaT1ca – an observational clinical cohort study to quantify liver health in surgical candidates for liver malignancies

**DOI:** 10.1186/s12885-018-4737-3

**Published:** 2018-09-12

**Authors:** Damian J. Mole, Jonathan A. Fallowfield, Timothy J. Kendall, Fenella Welsh, Scott I. Semple, Velicia Bachtiar, Matt Kelly, Stephen J. Wigmore, O. James Garden, Henry R. Wilman, Rajarshi Banerjee, Myrddin Rees, Michael Brady

**Affiliations:** 1 Medical Research Council Centre for Inflammation Research, University of Edinburgh, Edinburgh, UK; 2 Clinical Surgery, University of Edinburgh, Edinburgh, UK; 3 Division of Pathology, The University of Edinburgh, Edinburgh, UK; 4 Basingstoke and North Hampshire Hospital, Hampshire Hospitals NHS Foundation Trust, Hampshire, UK; 5 British Heart Foundation Centre for Cardiovascular Science, The University of Edinburgh, Edinburgh, UK; 6 Perspectum Diagnostics Ltd, 23-38 Hythe Bridge Street, Oxford, OX1 2ET UK; 7 Department of Life Sciences, University of Westminster, London, UK

## Abstract

**Background:**

Accurate assessment of liver health prior to undertaking resectional liver surgery or chemoembolisation for primary and secondary cancers is essential for patient safety and optimal outcomes. Liver*MultiScan*™, an MRI-based technology, non-invasively quantifies hepatic fibroinflammatory disease, steatosis and iron content. We hypothesise that Liver*MultiScan*™can quantify liver health prior to surgery and inform the risk assessment for patients considering liver surgery or chemoembolization and seek to evaluate this technology in an operational environment.

**Methods/Design:**

HepaT1ca is an observational cohort study in two tertiary-referral liver surgery centres in the United Kingdom. The primary outcome is correlation between the pre-operative liver health assessment score (Hepatica score - calculated by weighting future remnant liver volume by liver inflammation and fibrosis (LIF) score) and the post-operative liver function composite integer-based risk (Hyder-Pawlik) score.

With ethical approval and fully-informed consent, individuals considering liver surgery for primary or secondary cancer will undergo clinical assessment, blood sampling, and Liver*MultiScan*™multiparametric MRI before and after surgical liver resection or TACE. In nested cohorts of individuals undergoing chemotherapy prior to surgery, or those undergoing portal vein embolization (PVE) as an adjunct to surgery, an additional testing session prior to commencement of treatment will occur. Tissue will be examined histologically and by immunohistochemistry. Pre-operative liver health assessment scores and the post-operative risk scores will be correlated to define the ability of Liver*MultiScan*™to predict the risk of post-operative morbidity and mortality. Because technology performance in this setting is unknown, a pragmatic sample size will be used. For the primary outcome, *n* = 200 for the main cohort will allow detection of a minimum correlation coefficient of 0.2 with 5% significance and power of 80%.

**Discussion:**

This study will refine the technology and clinical application of multiparametric MRI (including Liver*MultiScan*™), to quantify pre-existing liver health and predict post-intervention outcomes following liver resection. If successful, this study will advance the technology and support the use of multiparametric MRI as part of an enhanced pre-operative assessment to improve patient safety and to personalise operative risk assessment of liver surgery/non-surgical intervention.

**Trial registration:**

This study is registered on ClinicalTrials.gov Identifier: NCT03213314.

**Electronic supplementary material:**

The online version of this article (10.1186/s12885-018-4737-3) contains supplementary material, which is available to authorized users.

## Background

An adequate level of liver function is essential for life. The liver is unique among the abdominal organs for the ability to regenerate following partial surgical resection (hepatectomy). Patients with pre-existing parenchymal liver disease have impaired liver regeneration and are at increased risk of postoperative morbidity and mortality after liver resection [1]. The ability to accurately assess liver health before surgery is a critical part of judging the safety of a planned hepatectomy, and is important for a well-informed discussion of the risks and benefits with individuals who are considering liver surgery. While the concept of having adequate liver volume and function is understood, there is no universally accepted method of functional liver assessment which has been applied to liver surgery. Because of the increased prevalence of non-alcoholic fatty liver disease (NAFLD) in the general population [2] – NAFLD now has a global prevalence of 25% [3] – it is estimated that 1 in 5 patients planned for liver resection have some degree of steatosis [4]. Even a mild degree of fat accumulation in the liver affects hepatocyte homeostasis and potentially impairs postoperative recovery after hepatectomy [5].

Here, we propose that multiparametric MRI including Liver*MultiScan*™ is one possible solution to this challenge. Liver*MultiScan*™is a non-invasive MRI-based technology shown to correlate with hepatic fibroinflammatory disease, steatosis and iron content [6], that can predict outcomes in patients with chronic liver disease [7]. In this study, we will use Liver*MultiScan*™as an additional quantitative measure of liver health prior to hepatectomy, typically done for colorectal liver metastases, or transarterial chemoembolization (TACE), typically done for hepatocellular carcinoma (HCC). In a small subset of patients in whom portal vein embolization (PVE) is performed prior to hepatectomy to promote hypertrophy of the FLR [8, 9], or in those receiving systemic anti-cancer therapy (chemotherapy), an additional pre-operative Liver*MultiScan*™assessment will be undertaken to detect FLR hypertrophy and any chemotherapy-associated liver injury prior to surgery. For patients with HCC, TACE is usually well-tolerated by people with healthy livers, but can precipitate liver decompensation in the context of pre-existing chronic liver disease [10]. We propose that Liver*MultiScan*™can also quantify liver health (and therefore risk of liver decompensation) after TACE.

In summary, this observational clinical cohort study aims to evaluate the technical utility of multiparametric MRI including Liver*MultiScan*™, to quantify pre-existing liver health and predict post-intervention outcomes following liver resection (with or without pre-operative chemotherapy or PVE) or TACE. The technology readiness level (TRL) of this study could be regarded as level 7 in the European Union scale, defined as, “system prototype demonstration in operational environment” [11]. If successful, this study will assist in refinement of the technology and provide proof-of-concept for multiparametric MRI to be progressed further in technological evaluation as part of an enhanced pre-operative assessment in patients to improve patient safety and to personalise the operative risk assessment of liver surgery/non-surgical intervention.

## Methods

### Study authorisations

This study has been reviewed as IRAS 223180 by the South East Scotland Research Ethics Committee 02, 17/SS/0049 who gave a favourable opinion. NHS Scotland R&D approval and NHS England HRA approval have been granted. The study sponsor is University of Edinburgh/NHS Lothian ACCORD.

### Study design

Observational cohort study.

### Setting

Two tertiary referral hepatobiliary surgery centres in the United Kingdom; (Royal Infirmary of Edinburgh and Basingstoke and North Hampshire Hospital).

### Timing

**Fig. 1:**
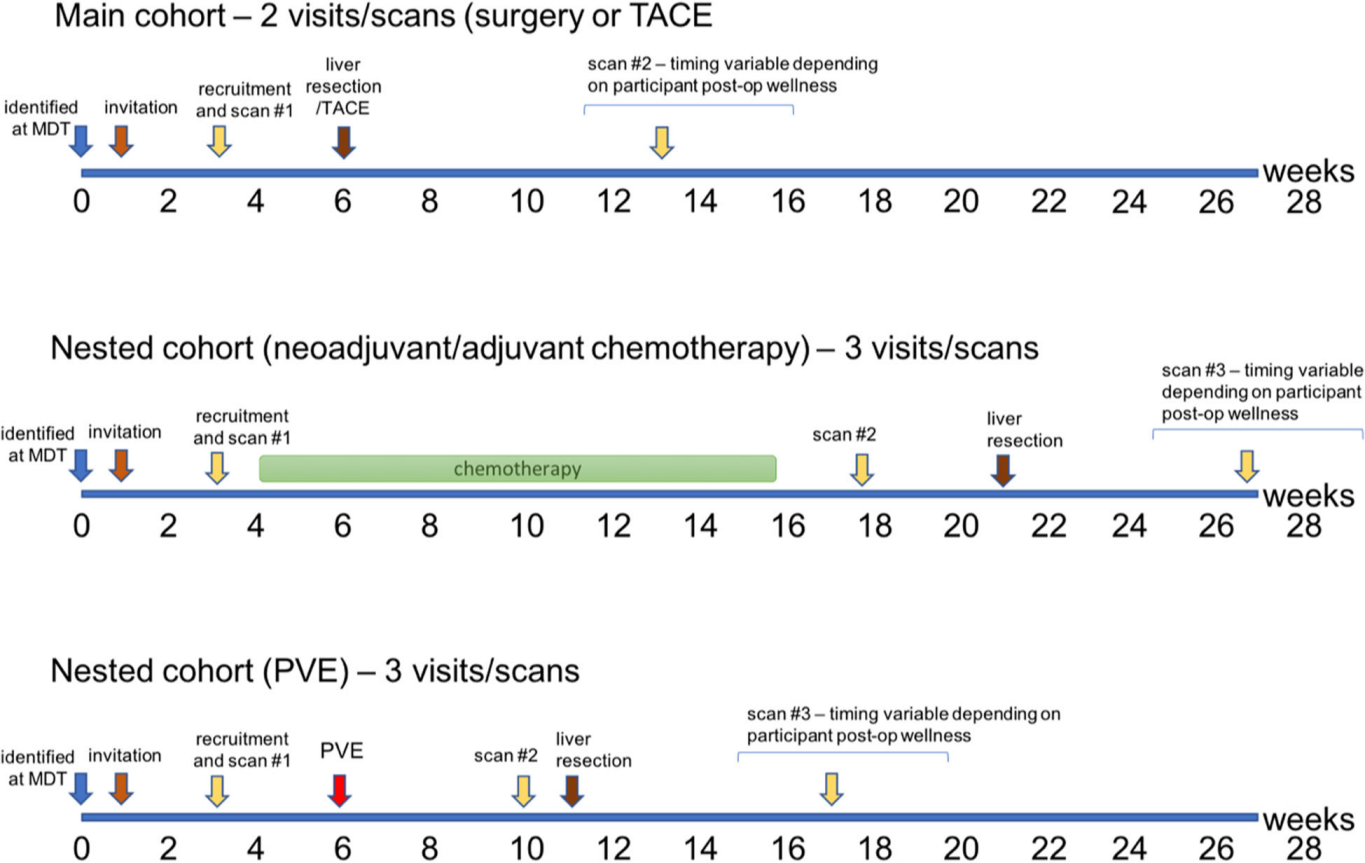
Timelines for participant visits for both the main cohort and the two nested cohorts (chemotherapy/portal vein embolization (PVE))

Recruitment is scheduled to begin on 1st August 2017 and continue until 31st January 2019. The general study outline and timelines are illustrated in Fig. 1.

### Intervention

No change in treatment, intervention or randomisation is proposed in the present study.

Eligible participants who have given informed consent will be asked to attend the local research facility for an initial study visit during which baseline clinical assessments, blood tests, and Liver*MultiScan*™MR imaging will be undertaken. Participants in the main study cohort will attend on a second occasion in the post-operative/post-TACE period (total 2 visits and scans). For participants who are enrolled in either of the nested study cohorts (i.e. those undergoing pre-operative chemotherapy or PVE as an adjunct to surgery), an additional scan visit will be required (total 3 visits and scans). An additional 6-month period will follow to complete the post-operative scans of the final recruited participants.

### Recruitment

All patients referred to the HPB multidisciplinary teams at each clinical site who are being considered for liver resection or TACE based on local NHS clinical care pathways will be eligible to participate. After an initial approach by a member of the direct clinical care team, interested patients will be contacted by a member of the research team by letter explaining the purpose and requirements of the study, and asked if they would like to participate. They will be invited to an initial screening appointment at which they can discuss the research further with the investigators and their eligibility to take part in the study will be confirmed.

#### Inclusion criteria

Male or female adult patients being considered for liver resection or TACE (for primary or secondary liver lesions).

#### Exclusion criteria

The following exclusion criteria will be adhered to:

i. Patients under the age of 18 years.
ii. Prisoners.
iii. Persons unable to have an MRI scan (including but not limited to claustrophobia, implanted metallic devices, metal foreign body)
iv. Adults with incapacity to provide informed consent
v. Non-provision of consent

### Participants

#### Consent

Potential participants will be identified as above. A member of the direct clinical care team looking after that patient (for example surgeon, oncologist, nurse specialist, GP) will make the initial contact with the patient in person or by phone (or post) and ask whether they are willing to be approached by the research team to consider participation. Those individuals who wish to consider participating will be sent an invitation letter and participant information sheet and invited to attend for a face-to-face meeting with a member of the research team where they will have the opportunity to ask questions before giving written informed consent and being recruited into the study. Recruitment and informed consent will be performed by the investigator/clinical research nurse. It will be made clear that the individual may withdraw from the study at any time without giving a reason and with no consequence to their current or future care. In addition, consent will be obtained to inform each participant’s GP and the Consultant in charge of their care.

#### Patient decision period

Potential participants who have been invited to participate will be given a period of up to 2 weeks (minimum 24 h) to read the relevant information sheets, to contact a member of the study team to get further information and ask questions, before they are invited to attend for a conversation and commence the process of fully-informed consent.

#### Withdrawal of participants

Participants will be withdrawn from the clinical research study in the following circumstances:

i. In cases of withdrawal of informed consent, where capacity to provide such consent exists.
ii. In cases of withdrawal of consent by the patient’s representative for adults who develop incapacity to continue to provide informed consent during the period of the study, for example due to critical illness.

Participants who wish to withdraw from the study will be given the option to permit ongoing use of data and samples which have already been collected, and/or future recording and usage of routinely collected clinical data and results. This will be clearly documented on the patient consent form.

### Measurements

#### Primary endpoint

To determine the ability of Liver*MultiScan*™to predict risk of post-operative morbidity and mortality by measuring the correlation between the pre-operative liver health assessment score (HepaT1ca score - calculated by weighting future remnant liver volume by liver inflammation and fibrosis (LIF) score) and the post-operative liver function composite integer-based risk (Hyder-Pawlik) score [12].

#### Secondary endpoints

- To compare Liver*MultiScan*™image interpretations with clinical outcomes after surgery or TACE in three domains: post-operative liver function, surgery-specific complication rate, and overall complication rate. The Clavien-Dindo classification (using all 5 grades) of morbidity will be used, in addition to free text descriptors of complications. Morbidity and mortality at 30 days and 90 days after surgery will be reported.
- To compare Liver*MultiScan*™image interpretations with histological findings in the resected liver specimen in four domains: fibrosis, inflammation, fat content and iron load
- To evaluate Liver*MultiScan*™image interpretations correlated with post-operative length of stay
- To evaluate Liver*MultiScan*™interpretations in the liver regeneration phase
- To identify prognostic biomarkers of liver health
- To evaluate whether Liver*MultiScan*™can add information as to the type of liver tumour
- To evaluate biliary pathology after chemotherapy and biliary regeneration after surgery with quantitative MRCP

#### Summary of clinical data to be collected

A full set of the data fields being collected has been exported from the data collection tool and is provided as Additional file 1.

At initial visit:

1. Demographic and general clinical background information,
2. Information regarding the primary liver lesion, and if liver metastases, information regarding the primary tumour,
3. Information relevant to background liver disease/function,
4. Information regarding the proposed surgery/intervention.

At interim visit: (in those having a second pre-operative scan after a period of adjuvant (to the colon primary), neoadjuvant or downstaging chemotherapy; or after PVE):

5. Information regarding the interim therapy received including adverse effects and complications.

At operation/TACE:

6. Details of surgery/TACE procedure, including weight of resected specimen.

At post-op visit:

7. Information regarding recovery from surgery or TACE procedure, including complications and post-op liver function.

#### Peripheral venous blood sampling

Blood samples will be obtained from each study participant on each visit and at the time of surgery for multiple routine and specialist assays, including genetic analysis on a maximum of four occasions over the course of the study.

#### MR imaging

A multiparametric MRI scan will be performed at each visit to measure lipid, iron load, and fibroinflammatory disease in the liver according to protocols in development at Perspectum Diagnostics Ltd. and undertaken at CRIC (Clinical Research Imaging Centre, QMRI, University of Edinburgh/Royal Infirmary of Edinburgh), or at Basingstoke and North Hampshire Hospital Department of Radiology. No intravenous contrast agents are used in Liver*MultiScan*™scans. For routine clinical care Primovist™ MRI will be performed subsequently at the same visit or on a separate occasion. The total duration of Liver*MultiScan*™research scans will be approximately 30 min.

#### Tissue sampling from the resected specimen

Tissue samples will be taken from liver resection specimens. Strict care will be taken not to compromise direct clinical care pathology reporting. Samples will include fresh and snap-frozen Tru-cut biopsies of the tumour and background liver parenchyma and formalin-fixed paraffin-embedded blocks of background liver and tumour tissue.

Samples of fresh background and tumour tissue will be analysed for standard laboratory assays, including for example for cell analysis, protein and gene expression, and will be subject to more advanced analyses e.g. integrative multiomics (including DNA and RNA sequencing, metabolomics and proteomics). Fresh liver tissue may be disrupted for primary cell cultures including for the development of in vitro assays, immortalized cell lines and organoids.

Samples of fixed background and tumour tissue will be analysed using histological and immunohistochemical techniques, but may also be subject to advanced analyses e.g. tissue expression mapping should protocols develop to allow this on fixed tissue in the future. Histology will be independently scored by a second observer. Tissue samples may be stored for at least 5 years from the date of collection.

### Data analysis plan

Standard statistical and data analysis packages, including R (Bell Laboratories, USA), MATLAB (MathWorks, USA) and Python (Python Software Foundation, USA) will be used. Evaluation of the novel diagnostic technologies will be performed according to the STARD framework (http://www.equator-network.org/reporting-guidelines/stard).

### Primary endpoint

*To determine ability of LiverMultiScan™ to predict risk of post-operative morbidity and mortality by measuring the correlation between the pre-operative liver health assessment score and the post-operative risk score*.

This analysis will measure the correlation between two continuous variables. The first variable will be the FLR volume weighted by the liver inflammation and fibrosis (LIF) score. This weighting will effectively decrease the FLR with increasing LIF score. The second variable will be the composite post-operative risk score as defined by Hyder and colleagues [12]. The distribution of both variables will be tested for Normality (e.g., with the Kolmogorov-Smirnov test) and the correlation calculated with the appropriate test (e.g., Pearson correlation if Normally-distributed; Spearman’s rank correlation if not, or non-linearly correlated).

#### Secondary endpoints

i) *To compare LiverMultiScan™ image interpretations with clinical outcome after surgery in three domains: post-operative liver function, surgery-specific complication rate and overall complication rate*.

This analysis will use a combination of statistical modelling techniques (e.g. logistic regression) and machine learning techniques (e.g., random forest and tensor flow) to identify the combination of MRI-derived measurements (e.g., LIF, proton density fat fraction, diffusion measurements) that best predict post-operative liver function, or classify patients based on complication rate. Derived models will be cross-validated using leave-*x*-out cross-validation to avoid over-fitting of the model and improve applicability to external populations.

The performance of classification models will be quantified by the area under the receiver operator curve (AUROC) along with the sensitivity, specificity, positive predictive value and negative predictive value for the most appropriate operating point. The statistical significance of any differences in MRI-derived measures of liver health between the different classes will be determined using the most appropriate test based on the Normality of the distributions and the number of groups being compared (e.g., t-test if comparing two Normally-distributed samples; ANOVA if comparing multiple Normally-distributed samples; Kruskal Wallis if comparing non-Normally-distributed samples). The performance of the regression models will be quantified using the appropriate correlation test (e.g., Pearson correlation if normally-distributed; Spearman’s rank correlation if not, or non-linearly correlated).

ii) *To compare LiverMultiScan™ image interpretations with histological findings in the resected specimen in four domains: fibrosis, inflammation, fat content and iron load*.
iii) *To evaluate LiverMultiScan™ image interpretations with post-operative length of stay*.
iv) *To evaluate LiverMultiScan™ image interpretations in the liver regeneration phase*.
v) *To identify additional biomarkers of liver health*.

This analysis will use a combination of statistical modelling techniques (e.g., logistic regression) and machine learning techniques (e.g., random forest and tensor flow) to identify those clinical and image-derived parameters being collected as part of the study that have utility in predicting liver health.

vi) *To evaluate whether LiverMultiScan™ can add information as to the nature of the liver tumour*.

This endpoint will be evaluated using the same analysis techniques described for the 1st secondary endpoint above to identify and calculate the utility of MRI-derived measurements for characterising liver tumour tissue.

vii) *To evaluate post-intervention cholangiopathy and biliary regeneration with quantitative MRCP*

This endpoint will use quantitative MRCP analysis technology to characterise the regeneration of biliary anatomy in the regenerating liver. This will be a descriptive analysis evaluating the change in intra-hepatic biliary volume, duct diameter and number of branches as the liver regenerates.

### Statistical analysis

#### Sample size

The sample size is pragmatic determined by the duration of the study and recruitment rate. We estimate the following sample size *n* = 100 per site (*n* = 200 total) which includes the nested cohort of *n* = 20 per site (*n* = 40 total). For the primary endpoint, this sample size would allow us to detect a minimum correlation coefficient of 0.2 with a 5% level of significance and power of 80% using a two-tailed test. Furthermore, this sample size will support the evaluation of the secondary endpoints, allowing us to account for any confounding factors (for example, post-operative length of stay may be influenced by lower discharge rates on weekends).

#### Benefit for participants

No direct benefit is expected for the participants. We envisage that the results of this study will benefit other patients being considered for liver surgery in the future, by developing new technology which improves patient safety, and society as a whole by advancing scientific knowledge of using imaging to quantify liver health. We believe that this study may lead to improvements in pre-operative planning which will prevent or ameliorate the impact of liver surgery for cancer. This will help to maximise the health, and life expectancy, of patients with liver cancers.

#### Potential risks and burdens for research participants

The participants will be subjected to peripheral venous blood sampling which may cause discomfort or pain. MRI scans are not expected to pose a risk to patients, in the absence of known contraindications. Patients will be assessed prior to the scan for any potential contraindications to the procedure such as mechanical heart valves, pacemakers, metallic prostheses and cochlear implants.

#### Dissemination

Results of the study will be presented at local, national and international medical meetings. The findings of the study will be published in peer reviewed medical/scientific journals and made open access on acceptance. If appropriate the results of the study will be disseminated by press releases by University of Edinburgh, Basingstoke and North Hampshire Hospital or Perspectum Diagnostics. Information may also be disseminated to the general public via public engagement and community outreach programmes.

## Discussion

Liver function can be regarded as the product of the total volume of hepatocytes and the health of those hepatocytes. After hepatectomy, a minimum level of liver function is necessary for survival. At present, that projected liver function is based almost entirely on the estimated FLR volume [13, 14], with most surgical teams adopting minimum thresholds for FLR of approximately 25% in normal livers, and 40% in cirrhotic livers [15]. Ideally, projected future liver function should be calculable as the product of the FLR volume and a continuous variable measure of hepatocyte function. At present, assessment of the FLR relies on computed tomography (CT) volumetric measurement combined with a crude estimate of hepatocyte function based on clinical judgment and gleaned from surrogate markers of liver function based on blood tests. Liver fat content (steatosis) can be measured on CT, or more accurately with magnetic resonance imaging (MRI) [16]. However, hepatic fibroinflammatory disease, which has been shown to correlate with overall and liver-related morbidity after resection [17], typically requires an invasive biopsy to diagnose accurately. Therefore, a preoperative non-invasive investigation that provides the estimated FLR and a validated measure of hepatocyte performance will be an important advance in the safety assessment of patients considering liver surgery.

## Additional file

Additional file 1: HepaT1ca study list of data fields as a database export. (PDF 67 kb)

## References

[1] van den Broek MA, Olde Damink SW, Dejong CH (2008). Liver failure after partial hepatic resection: definition, pathophysiology, risk factors and treatment. Liver Int.

[2] Harris R, Harman DJ, Card TR (2017). Prevalence of clinically significant liver disease within the general population, as defined by non-invasive markers of liver fibrosis: a systematic review. Lancet Gastroenterol Hepatol.

[3] Younossi ZM, Koenig AB, Abdelatif D (2016). Global epidemiology of nonalcoholic fatty liver disease-Meta-analytic assessment of prevalence, incidence, and outcomes. Hepatology.

[4] Veteläinen R, van Vliet A, Gouma DJ (2007). Steatosis as a risk factor in liver surgery. Ann Surg.

[5] Veteläinen R, Bennink RJ, van Vliet AK (2007). Mild steatosis impairs functional recovery after liver resection in an experimental model. Br J Surg.

[6] Banerjee R, Pavlides M, Tunnicliffe EM (2014). Multiparametric magnetic resonance for the non-invasive diagnosis of liver disease. J Hepatol.

[7] Pavlides M, Banerjee R, Sellwood J (2016). Multiparametric magnetic resonance imaging predicts clinical outcomes in patients with chronic liver disease. J Hepatol.

[8] Abdalla EK, Hicks ME, Vauthey JN (2001). Portal vein embolization: rationale, technique and future prospects. Br J Surg.

[9] Abulkhir A, Limongelli P, Healey AJ (2008). Preoperative portal vein embolization for major liver resection: a meta-analysis. Ann Surg.

[10] Garwood ER, Fidelman N, Hoch SE (2013). Morbidity and mortality following transarterial liver chemoembolization in patients with hepatocellular carcinoma and synthetic hepatic dysfunction. Liver Transpl.

[11] Horizon2020. HORIZON 2020 – WORK PROGRAMME 2014-2015. General Annexes. G. Technology readiness levels (TRL). 2015. Available from: http://ec.europa.eu/research/participants/data/ref/h2020/wp/2014_2015/annexes/h2020-wp1415-annex-g-trl_en.pdf. Accessed 30 July 2018.

[12] Hyder O, Pulitano C, Firoozmand A (2013). A risk model to predict 90-day mortality among patients undergoing hepatic resection. J Am Coll Surg.

[13] Kubota K, Makuuchi M, Kusaka K (1997). Measurement of liver volume and hepatic functional reserve as a guide to decision-making in resectional surgery for hepatic tumors. Hepatology.

[14] Abdalla EK, Adam R, Bilchik AJ (2006). Improving resectability of hepatic colorectal metastases: expert consensus statement. Ann Surg Oncol.

[15] Guglielmi A, Ruzzenente A, Conci S (2012). How much remnant is enough in liver resection?. Dig Surg.

[16] van Werven JR, Marsman HA, Nederveen AJ (2010). Assessment of hepatic steatosis in patients undergoing liver resection: comparison of US, CT, T1-weighted dual-echo MR imaging, and point-resolved 1H MR spectroscopy. Radiology.

[17] Reddy SK, Marsh JW, Varley PR (2012). Underlying steatohepatitis, but not simple hepatic steatosis, increases morbidity after liver resection: a case-control study. Hepatology.

