# Supplementary material for: Study protocol: HepaT1ca – an observational clinical cohort study to quantify liver health in surgical candidates for liver malignancies
Source: BMC Cancer. 2018 Sep 12;18:890. doi: 10.1186/s12885-018-4737-3 (PMC6136162; doi:10.1186/s12885-018-4737-3)
Supplement: Supplementary file 1 — HepaT1ca study list of data fields as a database export. (PDF 67 kb) [file 12885_2018_4737_MOESM1_ESM.pdf]

FIELD
Record ID
Event Name
Repeat Instrument
Repeat Instance
Data Access Group
Has the participant provided signed informed consent?
Is the patient being considered for liver resection / TACE (for primary or secondary liver lesions)?
Is the patient under the age of 18?
Is the patient a prisoner?

Is the patient unable to have an MRI scan (including but not limited to claustrophobia, implanted metallic devices, metal foreign body)?
Is the patient an adult with incapacity?
Is the participant eligible to participate in the study?
Complete?
Participant Cohort
Age (years)
Sex
Ethnicity
Please specify ethnicity
Expected pathology
Please specify expected pathology
Complete?
Ultrasound
Date of most recent scan
CT
Date of most recent scan
MRI
Date of most recent scan
MRCP
Date of most recent scan
PET
Date of most recent scan
Endoscopic ultrasound
Date of most recent scan
Laparoscopy +/- intraoperative USS
Date of most recent scan
Liver biopsy
Date of most biopsy
CEA
Alpha fetoprotein
CA 19-9
Complete?
ECOG score
Complete?
Does the participant have any comorbidities?

Ischaemic heart disease
Congestive Heart Failure
Valvular Heart Disease
Please specify
Chronic Kidney Disease
Please select stage
Cerebrovascular Disease (e.g. TIA, stroke, CVA)
Peripheral Vascular Disease
Chronic Respiratory Disease
Asthma
COPD
Other Chronic Respiratory Disease
Please specify
Diabetes
Diabetes type
Please specify
Complete?
Known liver disease
If yes, aetiology
Aetiology - other
If yes, second aetiology
Second aetiology - other
Pre-operative diagnosis of cirrhosis
Complete?

Is the participant part of a nested cohort requiring a second pre-operative scan?
Please specify cohort
Complete?
Blood sample date
WBC (x10^9/L)
RBC (x10^12/L)
Hb (g/L)
HCT
MCV (fL)
PLT (x10^9/L)
Neutrophils (x10^9/L)
Lymphocytes (x10^9/L)
Fibrinogen (g/L)
APTT (Seconds)
PT (seconds)
INR
EDTA collected (plasma analysis)
Gel clot-activator collected (serum analysis)
PaxGene DNA collected (DNA extraction)
PaxGene RNA collected (Total RNA extraction)
Complete?
Sodium (mmol/L)
Potassium (mmol/L)

Urea (mmol/L)
Creatinine (umol/L)
Glucose (mmol/L)
Bilirubin (umol/L)
AST (U/L)
ALT (U/L)
Alk phos (U/L)
GGT (U/L)
Calcium (mmol/L)
Albumin (g/L)
Ferritin (ug/L)
Transferrin (g/L)
Serum Iron (umol/L)
CRP (mg/L)
Complete?
Ascites
Encephalopathy
Albumin classification
Bilirubin classification
INR classification
Ascites classification
Encephalopathy classification
Total score
Class
No. of previous decompensation episodes
Meld score
Complete?
Spleen size recorded (USS)
Spleen diameter (cm)
Varices
Banding Program
Other evidence of portal hypertension
Complete?
Is the participant taking any medications?
Complete?
Any change in medication since last visit?
Complete?
Alcohol consumed
What is the average weekly intake?
Participant abstinent for..
Coffee Intake
If yes, specify
Cups per day
Current Status
Pack years
Complete?
Colorectal Primary Tumour?
Site of colorectal primary

Colorectal primary resected
Date of resection
Operation type
Operation type - other, please specify
Was colon op laparoscopic
Endoscopic mucosal resection/polypectomy
Colonic stent
Tumour type - adenocarcinoma
If no or variant, please specify
Differentiation by predominant area
TMN (tumour) stage
TMN (node) stage
TMN (metastasis) stage
Duke's classification
Colon resection status
K-ras status
N-ras status
B-Raf status
Mismatch repair status
Pre-op (colon surgery) chemo
Date chemo commenced
Date of last chemo administration
Number of cycles planned
Number of cycles completed
Total duration of chemo (months)
Chemotherapy Regimen
Chemotherapy regimen - other
Grade 3 or 4 toxicities?
Pre-op radiotherapy
Complete?
Pre-operative (liver surgery) chemotherapy
Is this the same chemotherapy as colorectal primary tumour?
Indication/intent
Date chemo commenced
Date chemo finished
Number of cycles planned
Number of cycles completed
Chemotherapy Regimen
Other chemotherapy regimen - please specify
Response (N.B. local investigator assessed not RECIST criteria)
Grade 3 or 4 toxicities?
Date of last chemotherapy (prior to 2nd MRI scan or surgery)
Pre-op portal vein embolization
Pre-op portal vein embolization - please provide date
Pre-PVE Future Liver Remnant Volume (ml)
Pre-op biliary drainage
Pre-op biliary drainage - please provide date
Date of pre-op hepatic vein embolization

Complete?
Participant due to undergo TACE?
Albumin < 36 g/dL?
AFP >400 ng/mL?
Bilirubin >17 umol/L?
Max. tumour diameter >7 cm
HAP score
HAP class
Complete?
Heart rate (beats per min)
Blood pressure (sitting) mmHg Systolic
Blood pressure (sitting) mmHg Diastolic
Respiratory rate (breaths per min)
Tympanic temperature (C)
Weight (kg to the nearest 0.1 kg)
Height (cm)
Body mass index (kg/m2)
Complete?
Did operation go ahead?
Date of operation
Operation type
Operation type - other
Please give reason
ASA grade
Resection type
If anatomic resection please specify:
If anatomic resection, predicted SPECIMEN volume (cm^3)
Total number of atypical incisions
Seg 1
Seg 2
Seg 3
Seg 4
Seg 5
Seg 6
Seg 7
Seg 8
Predicted total liver volume on imaging (cm^3)
Predicted functional liver remnant volume (FLRV) (cm^3)
Atypical excision(s)
Total number of Atypical excision(s)
Surgical access (abdominal)
Vascular resection (IVC/PV)
Additional extrahepatic procedure
Please specify additional extrahepatic procedure
Back table sample taken (tumour)
Back table sample taken (background liver)
Pringle
Pringle Total Time (mins)

Fibrin glue
TachoSil
Fibrin pad
Operation duration - knife to skin to op end (mins)
Estimated Intraoperative blood loss (mL)
Intraoperative blood transfusion (units)
Total blood transfusion (units)
Abdominal drain placed at surgery
Duration of drain placed at surgery (days)
Abdominal drain placed percutaneously after surgery
Duration of perc drain (days)
Epidural
Vasopressor use
Vasopressor use (days)
Haemofiltration/dialysis
Haemofiltration/dialysis (days)
Date of Admission to hospital
Date of discharge or death
Number of ICU admissions
Number of HDU admissions
Total Duration of ICU stay (days)
Total duration of HDU stay (days)
Complete?
Participant undergone TACE
Child Pugh increase (following TACE)
AST increase >25% from baseline
Radiologic tumour response
ART score
ART score group Low is 0 - 1.5 points High is >= 2.5 points
Complete?
Did the patient have major liver complications?
Bleed
return to theatre
Liver failure
percutaneous drainage of collection
anastamotic leak requiring return to theatre
other
Other major liver complications - please specify
Clavien Dindo
INR
Serum Bilirubin
Serum Creatinine
Encephalopathy grade (West Haven classification)
Liver flap
INR
Serum Bilirubin
Serum Creatinine
Encephalopathy grade (West Haven classification)

Liver flap
INR
Serum Bilirubin
Serum Creatinine
Encephalopathy grade (West Haven classification)
Liver flap
INR
Serum Bilirubin
Serum Creatinine
Encephalopathy grade (West Haven classification)
Liver flap
INR
Serum Bilirubin
Serum Creatinine
Encephalopathy grade (West Haven classification)
Liver flap
INR
Serum Bilirubin
Serum Creatinine
Encephalopathy grade (West Haven classification)
Liver flap
Complete?
Is the participant still alive?
If no, date of death
Number of reported non-Liver complications
Clavien Dindo
Number of liver lesions
Seg 1
Seg 2
Seg 3
Seg 4
Seg 5
Seg 6
Seg 7
Seg 8
Known vascular invasion
Complete?

Clotting factors given to correct INR anytime up to and including day 5
Elevated INR and increasing between day 4 and day 5
Elevated bilirubin and increasing between day 4 and day 5

Abnormal liver blood tests but no change in management (ISGLS grade A)
Abnormal liver blood tests requiring non-invasive change in management (ISGLS grade B)

Abnormal liver blood tests requiring invasive change in management (ISGLS grade C)
Post-operative sepsis

Antibiotic duration (days)
Blood cultures +ve at any stage post-op
Organism
Abdominal drain culture fluid +ve
Drain culture organism
Other culture site positive - please state site
Other culture site organism
Complete?
Diagnosis
Diagnosis - other, please specify
If HCC
If HCC - other, please specify
If intrahepatic cholangiocarcinoma
Specimen 1 length (mm)
Specimen 1 width (mm)
Specimen 1 depth (mm)
Specimen 2 length (mm)
Specimen 2 width (mm)
Specimen 2 depth (mm)
Specimen 3 length (mm)
Specimen 3 width (mm)
Specimen 3 depth (mm)
Lesion 1 size (mm)
Lesion 2 size (mm)
Lesion 3 size (mm)
Lesion 4 size (mm)
Lesion 5 size (mm)
>5 lesions, size of largest (mm)
Completeness of resection
Macroscopic distance from hepatic resection margin (mm)
Tumour cells present at margin (microscopic)
If margin is clear (microscopic), is clearance > 10 mm
If no, minimum distance to margin (mm)
Histological Grade
Mucin Score
Special Stains / IHC (e.g. Ki67)
If yes, please specify
Complete?
Background liver assessed?
Fat % core area (H&E)
Fat % (oil-red O quantitation)
Histological diagnosis of steatohepatitis
Kleiner (Brunt) steatosis score
NAFLD activity score (Hepatocyte ballooning)
NAFLD activity score (Lobular inflammation)

NAFLD activity score (NAS on H&E) (see appendix for scoring system)
NASH-CRN fibrosis score

SAF ballooning score, with components
SAF inflammation score
Scheuer siderosis score
Modified Ishak fibrosis score
Chemo - perivenular sinusoidal dilatation
Chemo - portal liver injury
Complete?
Date of scan
Liver T1 - Mean (ms)
Liver T2* - Mean (ms)
Liver Iron - mean (mg/g)
Liver IDEAL PdFF - Mean (%)
Liver cT1 - Mean (ms)
Liver cT1 - Mode (ms)
LIF score - Mean
LIF 0-1 (%)
LIF 1-2 (%)
LIF 2-3 (%)
LIF 3-4 (%)
Spleen size - diameter (cm)
Spleen volume (cm^3)
Splenic T1 - Mean (ms)
Splenic T2* - Mean (ms)
Splenic cT1 - Mean (ms)
Splenic cT1 - Mode (ms)
Biliary tree volume (ml)
Number of branches
Biliary tree desciption
ADC - Mean (mm^2/s)
D - Mean (mm^2/s)
D* - Mean (mm^2/s)
F - Mean (%)
Liver volume (ml)
Predicted future liver remnant (%)
Adverse Event description
Start date (day)
Start date (month)
Start date (year)
SAE
Severity
Causality
Expectedness
Date of assessment
Initials of delegated clinician
Outcome
Resolved date
Complete?
Date of change of status / withdrawal

Participant deceased
Date of death
Who is withdrawing the participant from the trial?
Reason for withdrawal (please select one)
If Other, please specify
Withdrawal status
Complete?
